# Clinical outcomes and dialysate calcium concentrations in Chinese patients on maintenance hemodialysis: a retrospective cohort study

**DOI:** 10.1080/0886022X.2025.2536194

**Published:** 2025-08-04

**Authors:** Yurong Pan, Yunhan He, Yunyan Murong, Juan Xu, Wenqin Yang, Wenyan Guo, Jianlin Jian, Haiwen An, Yanlin Li, Qingxiu Huang, Linna Liu

**Affiliations:** ^a^ Department of Nephrology, Zhongshan Hospital of Traditional Chinese Medicine Affiliated to Guangzhou University of Traditional Chinese Medicine, Zhongshan, China; ^b^ School of Medicine, Nanjing University of Chinese Medicine, Nanjing, China

**Keywords:** Maintenance hemodialysis, dialysate calcium concentration, mortality, matched-pair analysis

## Abstract

**Background:**

The appropriate concentration of dialysate calcium (DCa) for maintenance hemodialysis (MHD) patients remains a subject of ongoing debate. The relationship between DCa concentration and patient outcomes is not yet well established. This study aimed to evaluate the impact of DCa concentration on mortality and major adverse cardiovascular and cerebrovascular events (MACCEs) in Chinese patients undergoing MHD.

**Methods:**

A retrospective matched cohort study was conducted, analyzing data from hemodialysis (HD) patients at our center over the past five years. Each patient in the low DCa group was matched with a counterpart from the mid DCa group. Outcomes, including mortality, MACCE rates, fracture rates, and hospitalization rates, were compared between the two groups.

**Results:**

The study included 924 MHD patients, of whom 101 low DCa patients were matched with 101 mid DCa patients. In the matched-pair analysis, no significant difference was observed in all-cause mortality between the low DCa and mid DCa groups (3.5/100 vs. 4.7/100 patient-years). However, the low DCa group exhibited lower cumulative rates of MACCEs, hospitalization, and fracture incidence compared with the mid DCa group (7.3/100 vs. 20.7/100 patient-years, 21.5/100 vs. 34/100 patient-years, and 1.6/100 vs. 3.9/100 patient-years, respectively). In addition, the adjusted hazard ratio (HR) for the occurrence of first MACCE in the low DCa group compared with the mid DCa group was 0.47 (95% CI: 0.24–0.91).

**Conclusions:**

These findings suggest that low DCa concentrations are associated with reduced rates of MACCEs, hospitalization, and cumulative fracture incidence in MHD patients relative to mid DCa concentrations.

## 1. Introduction

Mineral and bone disorders associated with chronic kidney disease (CKD-MBD) are common complications in individuals with end-stage renal disease (ESRD) [1]. These disorders are characterized by abnormalities in calcium, phosphorus, and intact parathyroid hormone (iPTH) levels [2]. Dysregulated mineral metabolism can lead to vascular calcification, bone disease, and increased mortality risk [3,4]. Maintenance hemodialysis (MHD) further exacerbates disturbances in mineral metabolism [1], with the calcium concentration in the dialysate being a critical factor influencing calcium balance. Dialysate calcium (DCa) concentration has been shown to significantly impact all-cause mortality and the prevalence of cardiovascular diseases in ESRD patients [4–6].

Although calcium phosphate binders, vitamin D, and other medications can be combined with an appropriate DCa concentration to improve calcium, phosphorus, and parathyroid hormone (PTH) levels in HD patients, the optimal DCa for individuals undergoing long-term MHD remains controversial [7]. The Kidney Disease: Improving Global Outcomes (KDIGO) guidelines recommend a DCa range of 1.25–1.50 mmol/L (2.5–3.0 mEq/L) for dialysis patients, but the choice should be tailored to the patient’s CKD-MBD status [8]. Higher DCa concentrations often lead to calcium overload, cardiovascular calcification, and suppression of iPTH secretion. Conversely, low DCa concentrations increase the risk of hypocalcemia, severe secondary hyperparathyroidism, and accelerated bone loss. Both excessively high and low DCa levels are associated with a greater incidence of adverse outcomes [8,9]. The evidence on the impact of DCa concentration is inconsistent. A prospective Korean cohort study demonstrated that elevated DCa is significantly associated with increased all-cause mortality and hospitalization rates in MHD patients [7]. In contrast, research by the Japan Society for Dialysis Therapy found no significant correlation between DCa concentration and overall mortality rates [10,11]. Supporting this, Kamei et al. through a systematic review and meta-analysis, reported no substantial differences in cardiovascular calcification or mortality between low and high DCa concentrations. Variations in patient selection, cardiovascular disease burden, and differing DCa standards may explain these conflicting findings [12].

The ideal DCa level has yet to be agreed upon, especially in terms of its effects on clinical outcomes among the Chinese MHD population, which require further investigation. To contribute to this ongoing debate, we evaluated the impact of DCa on all-cause mortality and major adverse cardiovascular and cerebrovascular events (MACCEs) in Chinese patients undergoing MHD. A matched-pair analysis was employed to mitigate bias associated with the nonrandom treatment assignments inherent in retrospective studies.

## 2. Materials and methods

### 2.1. Ethics statement

This study received approval from the Institutional Review Board of Zhongshan Hospital of Traditional Chinese Medicine, affiliated with Guangzhou University of Traditional Chinese Medicine (Approval No. 2024ZSZY-LL-KY-280). This study was conducted in compliance with the principles outlined in the Declaration of Helsinki and was granted a waiver for informed consent owing to its retrospective design.

### 2.2. Study design, setting, and cohorts

This retrospective observational matched cohort study analyzed maintenance hemodialysis (MHD) patients enrolled by our center in the Chinese National Renal Data System (CNRDS) between January 1, 2019, and December 31, 2023. The cohort primarily comprised patients using either low DCa (1.25 mmol/L) or mid DCa (1.5 mmol/L). The dialysate composition prescribed on the day of enrollment was recorded, and patients were required to have used the specified calcium dialysate concentration for at least three months. The inclusion criteria were as follows: initiation or ongoing maintenance hemodialysis treatment (three times a week); no previous kidney transplant; adult (aged ≥ 18 years) dialysis patients; having complete baseline information. Patients were excluded for the following reasons: concurrent HD and peritoneal dialysis, dialysis follow-up duration of less than three months, history of renal transplantation, or missing information on dialysis calcium concentration. A total of 924 MHD patients were eligible for enrollment, including 113 patients in the low DCa group and 811 patients in the mid DCa group.

To minimize imbalance and selection bias between the low DCa and mid DCa groups, a matched-pair analysis was performed at a 1:1 ratio. Patients were matched based on the following criteria: date of first dialysis (within one-year categories), age (within five-year categories), and sex.

To calculate survival time, the matched mid DCa patients were assigned the calendar date corresponding to the first use of low-calcium prescription dialysis by their respective matched low DCa counterparts. This date is referred to as the start date (d). Matched mid DCa patients were required to be alive on this date. The follow-up period ended upon the occurrence of one of the following events: death, a censoring event (such as a switch to an alternative dialysis modality, kidney transplantation, or transfer to another dialysis center), or the conclusion of the study follow-up on December 31, 2023.

### 2.3. Data collection

Baseline demographic characteristics, comorbidities, and laboratory parameters were gathered from the hospital’s inpatient system and cross-referenced with data from the CNRDS for accuracy. Demographic details included date of birth, sex, type of initial vascular access (arteriovenous fistula, tunneled central catheter, or arteriovenous graft), primary kidney disease, and date of first dialysis. Comorbidities at baseline were identified based on the International Classification of Diseases, 9th and 10th Revision (ICD-9 and ICD-10) codes in the medical records. The Charlson Comorbidity Index (CCI) was calculated according to the method described by Quan et al. [13]. Laboratory parameters included serum hemoglobin, albumin, creatinine, blood urea nitrogen, total cholesterol, calcium, phosphorus, and iPTH. The estimated glomerular filtration rate (eGFR) was calculated using the Chronic Kidney Disease Epidemiology Collaboration (CKD-EPI) equation prior to the initiation of dialysis. [14]^.^

### 2.4. Outcomes and exposures

The primary outcome of this study was all-cause mortality. Secondary outcomes included MACCEs, fractures, and hospitalizations. In this study, MACCEs were specifically defined as cerebral hemorrhage, stroke, heart failure, myocardial infarction, unstable angina, peripheral vascular events, and sudden death [15]. All outcome data were retrospectively collected from the CNRDS and the hospital’s inpatient records.

### 2.5. Statistical analysis

All statistical analyses were performed using IBM SPSS (version 25.0) and GraphPad Prism (version 8.0). Data with a normal distribution are presented as the mean ± standard deviation, while data without a normal distribution are presented as the median with range. Categorical variables are expressed as numbers (percentages). We used the standardized mean difference (SMD) to assess the balance of each baseline covariate between the groups before and after the matched-pair analysis, with an SMD greater than 0.1 indicating covariate imbalance. Kaplan–Meier survival curves were generated to evaluate the effects of low and medium calcium dialysate on mortality, first fracture event, and first MACCE occurrence. The time scale was defined as the interval from the start date (d) to the end of the follow-up. Differences were assessed using the log-rank test. Univariable and multivariable Cox proportional hazards regression models were used to calculate hazard ratios (HRs) with 95% confidence intervals (CIs) for death, first MACCE, first fracture and first hospitalization between the low DCa and mid DCa groups. Additionally, the multiplicative model was used to assess the impact of the interaction between dialysis calcium concentration and iPTH levels on the outcome of MACCEs. A p-value < 0.05 was considered statistically significant. To control for baseline iPTH levels, the 2017 KDIGO guidelines were followed, categorizing patients into two groups based on the optimal cutoff value: iPTH < 300 pg/mL and iPTH ≥ 300 pg/mL [8]. Kaplan-Meier analysis and univariable and multivariate Cox proportional hazards regression models were used to stratify and compare of mortality and the first MACCE occurrence between the two groups of patients after matching. Moreover, the interaction between dialysis calcium concentration and iPTH was evaluated in both subgroups.

## 3. Results

### 3.1. Patients and data

**Figure 1.**
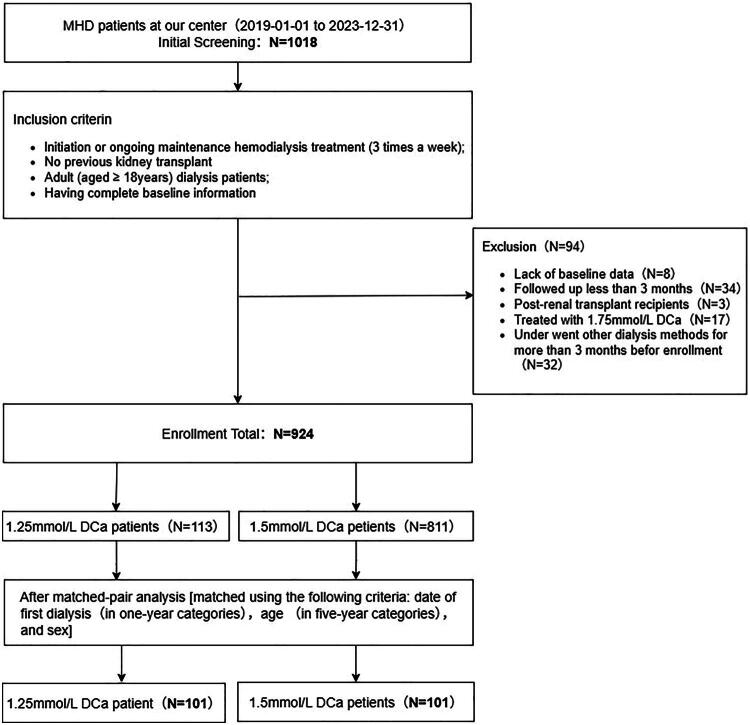
Study profile. MHD: maintenance hemodialysis; DCa: dialysate calcium.

The study cohort profile is shown in Figure 1. A total of 1,018 patients were registered in the CNRDS by our center during the study period. Ninety-four patients were excluded for the following reasons: concurrent HD and peritoneal dialysis (*n* = 32), follow-up duration of less than three months (*n* = 34), missing baseline data (*n* = 8), and renal transplantation (*n* = 3). In addition, 17 patients receiving 1.75 mmol/L dialysis calcium were excluded. Of the 924 patients who entered the follow-up, 113 received 1.25 mmol/L dialysis calcium (low DCa) and 811 received 1.5 mmol/L dialysis calcium (mid DCa). Ultimately, 101 patients with low DCa were successfully matched with 101 patients with mid DCa. However, 710 patients with mid DCa and 12 patients with low DCa were excluded from the analysis because they did not meet the inclusion criteria.

### 3.2. Baseline characteristics of patients

Among the 924 MHD patients, 12.2% (113 patients) were categorized into the low DCa group, while the remaining 87.8% (811 patients) were assigned to the mid DCa group. Baseline characteristics are presented in Table 1. Before matching, the low DCa group had a longer baseline dialysis duration [16.5 ± 34.0 vs. 62.2 ± 56.6 months, *SMD = 1.22*] and a lower CCI score [3.6 ± 1.4 vs. 3.2 ± 1.2, *SMD = 0.29*] compared with the mid DCa group. In addition, the prevalence of congestive heart failure and peripheral vascular disease was notably lower in the low DCa group. Regarding the primary causes of ESRD, glomerulonephritis was significantly more common in the low DCa group (68.1%). In laboratory parameters, the low DCa group exhibited lower levels of blood urea nitrogen, serum creatinine, cholesterol, serum albumin, hemoglobin, corrected calcium, phosphorus, iPTH, and low-density lipoprotein, with the most pronounced difference observed in iPTH levels. After matched-pair analysis, there were no significant differences between the low DCa and matched mid DCa groups in terms of demographic characteristics, causes of ESRD, and vascular access (Table 1).

**Table 1.** Baseline characteristics of patients in the 1.25 mmol/L and 1.5 mmol/L DCa groups before and after matching.

Characteristics	Before Matching			After Matching		
	All Mid DCa Group 1.5 mmol/L (*n* = 811)	All Low DCa Group 1.25 mmol/L (*n* = 113)	SMD	Matched Mid DCa Group 1.5 mmol/ (*n* = 101)	Matched Low DCa Group 1.25 mmol/L (*n* = 101)	SMD
**Demographic data**						
Female (n)	518 (63.9%)	64 (56.6%)	0.15	55 (54.5%)	55 (54.5%)	0.00
Male (n)	293 (36.1%)	49 (43.4%)		46 (45.5%)	46 (45.5%)	
**Age (years)**	58.3 ± 14.4	55.6 ± 14.2	0.18	57.0 ± 13.9	55.6 ± 14.3	0.09
Dialysis vintage (months)	16.5 ± 34.0	62.2 ± 56.6	1.22	56.1 ± 47.7	57.4 ± 46.7	0.07
**Causes of ESRD**			0.27			0.02
Glomerulonephritis (n)	446 (55.0%)	77 (68. 1%)		70 (69.3%)	69 (68.3%)	
Diabetic nephropathy (n)	310 (38.2%)	33 (29.2%)		23 (22.8%)	32 (31.7%)	
Other/unknown (n)	55 (6.8%)	3 (2.7%)		8 (7.9%)	3 (3.0%)	
**Comorbidities**						
Charlson Comorbidities Index	3.6 ± 1.4	3.2 ± 1.2	0.29	3.5 ± 1.5	3.4 ± 1.5	0.08
Diabetes (n)	313 (38.6%)	33 (29.2%)	0.20	34 (33.7%)	32 (31.7%)	0.04
Hypertension (n)	749 (92.4%)	106 (93.8%)	0.06	93 (92. 1%)	95 (94. 1%)	0.08
Coronary artery disease (n)	132 (16.3%)	13 (11.5%)	0.14	14 (13.9%)	11 (10.9%)	0.09
Cerebrovascular disease (n)	236 (29. 1%)	30 (26.5%)	0.06	26 (25.7%)	27 (26.7%)	0.02
Peripheral vascular disease (n)	258 (31.8%)	25 (22.1%)	0.22	30 (29.7%)	23 (22.8%)	0.16
Congestive heart failure (n)	159 (19.6%)	12 (10.6%)	0.25	19 (18.8%)	10 (9.9%)	0.26
Cancer (non-skin) (n)	34 (4.2%)	7 (6.2%)	0.09	5 (5.0%)	5 (5.0%)	0.00
**Laboratory tests**						
BUN (mmol/L)	27.1 ± 8.7	26.9 ± 7.2	0.02	27.5 ± 7.7	27.1 ± 6.9	0.05
Serum creatinine (μmol/L)	966.3 ± 316.7	1071.3 ± 237.8	0.34	1000.5 ± 278.0	1070.3 ± 244.0	0.27
Cholesterol (mmol/L)	4.2 ± 1.1	4.5 ± 1.0	0.27	4.4 ± 1.2	4.5 ± 1.0	0.09
Plasma albumin (g/dL)	3.8 ± 0.4	3.9 ± 0.3	0.26	38.3 ± 3.6	39.2 ± 3.0	0.05
Hemoglobin (g/dL)	98.9 ± 21.3	107.7 ± 14.3	0.43	104.4 ± 18.0	106.7 ± 14.1	0.14
Serum calcium (mg/dL), albuimin-Corrected	8.8 ± 0.9	9.4 ± 1.0	0.65	8.8 ± 0.8	9.4 ± 1.0	0.66
Serum phosphorous (mmol/L)	2.0 ± 0.7	2.2 ± 0.9	0.29	2.1 ± 0.7	2.2 ± 0.6	0.15
Serum iPTH (pg/mL)	376.2 ± 396.5	588.6 ± 449.7	0.52	421.1 ± 448.2	573.7 ± 445.3	0.34
Serum LDL cholesterol (mmol/L)	2.2 ± 0.8	2.4 ± 0.8	0.25	2.3 ± 0.9	2.4 ± 0.9	0.11
**Vascular access**			0.18			0.00
AVF (n)	702 (86.6%)	104 (92%)		92 (91. 1%)	92(91.1%)	
TCC (n)	98 (12. 1%)	7 (6.2%)		8 (7.9%)	7(6.9%)	
AVG (n)	11 (1.4%)	2 (1.8%)		1 (1.0%)	2(2.0%)	
**Medication use**						
Noncalcium binder (n)	158 (19.5%)	53 (46.9%)	0.59	40 (39.6%)	45 (44.6%)	0.10
Calcium binder (n)	155 (19.1%)	27 (23.9%)	0.12	31 (30.7%)	26 (25.7%)	0.11
Vitamin D (n)	204 (25.2%)	62 (54.9%)	0.62	46 (45.5%)	54 (53.5%)	0.16
Cinacalcet (n)	157 (19.4%)	57 (50.4%)	0.67	32 (31.7%)	36 (35.6%)	0.08

SMD: standard mean difference; DCa, dialysate calcium concentration; BUN, blood urea nitrogen; ESRD, end-stage renal disease; iPTH, intact-parathyroid hormone; eGFR, estimated glomerular filtration rate; LDL, low-density lipoprotein; TCC: tunneled cuffed catheter; AVF: arteriovenous fistulas; AVG: Arteriovenous graft.

### 3.3. Patient-level outcomes

#### 3.3.1. Mortality

**Figure 2.**
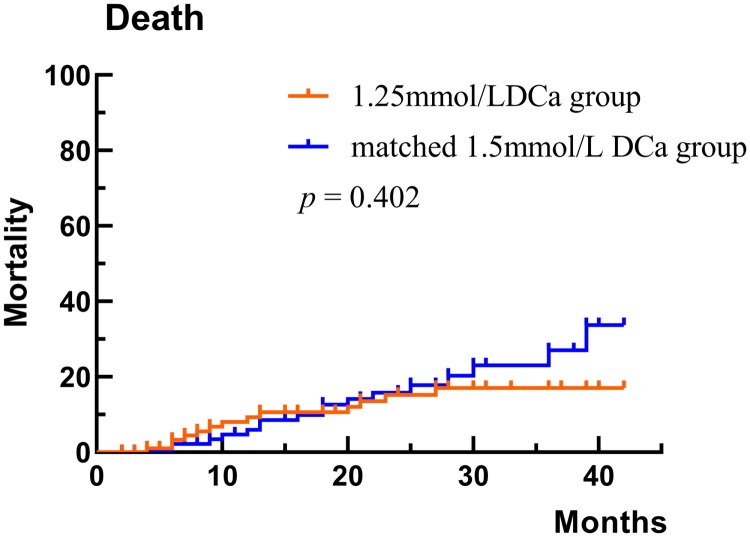
Kaplan-Meier Survival for the 1.25mml/L DCa group and matched 1.5 mmol/L DCa group patients. DCa: dialysate calcium.

After matching, the median follow-up period was 45 months for the matched mid DCa group and 41 months for the low DCa group. A total of 13 patients (12.9%) in the low DCa group died, with the causes of death categorized as follows: 2 deaths due to MACCEs, 8 from infections, and 3 from other or unknown causes. In comparison, 17 patients (16.8%) in the matched mid DCa group died, with causes of death distributed as follows: 4 from MACCEs, 5 from infections, and 8 from other or unknown causes. The causes of death did not differ significantly between the two groups (*p* = 0.20, Table 3). As shown in Table 2, the exposure-adjusted mortality rate was 3.5 per 100 patient-years in the low DCa group and 4.7 per 100 patient-years in the matched mid DCa group (*p* = 0.18). In addition, no significant association was observed between dialysis calcium concentration and all-cause mortality rate in both univariable and multivariable Cox proportional hazards analyses (Table 4). Similarly, the Kaplan-Meier survival curve demonstrated no significant difference in survival between the low DCa and matched mid DCa groups (log-rank test *p* = 0.402, Figure 2).

**Table 2.** Patient-level outcomes of 1.25 mmol/L DCa group and matched 1.5 mmol/L DCa group.

	1.25 mmol/L DCa group (*n* = 101, 367.8 patient-years)		1.5 mmol/L DCa group (*n* = 101, 361.8 patient-years)		
Events	No. of Events	Exposure-Adjusted Rate (per 100 patient-year)^#^	No. of Events	Exposure-Adjusted Rate (per 100 patient-year)^#^	P*
Death	13	3.5	17	4.7	0.18
MACCEs	27	7.3	75	20.7	**<0.001**
Hospitalization	79	21.5	123	34	**<0.001**
Fracture	6	1.6	14	3.9	**0.003**

MACCEs, main adverse cardiovascular and cerebrovascular events; DCa, dialysate calcium concentration.

*p values were calculated for the exposure-adjusted incidence rate.

^#^The exposure-adjusted rate was calculated as 100 times the total number of events divided by the total number of patient-years of exposure.

Bold values indicates significant statistical differences.

**Table 3.** Cause of death in 1.25 mmol/L DCa and 1.5 mmol/L DCa patients after matching.

	1.25 mmol/L DCa Group (13 Deaths)	1.5 mmol/L DCa Group (17 Deaths)	*P*
MACCEs (n)	2 (15.4%)	4 (23.5%)	0.20
Infectious diseases (n)	8 (61.5%)	5 (29.4%)	
Others or unknown (n)	3 (23.1%)	8 (47.1%)	

DCa, dialysate calcium concentration; MACCEs, main adverse cardiovascular and cerebrovascular events.

**Table 4.** Risk factors for mortality and the occurrence of first MACCE assessed by Cox regression model after matching.

	Death				MACCEs			
	Univariable		Multivariable		Univariable		Multivariable	
Variables	Crude HR (95% CI)	*P*	Adjusted HR (95% CI)	*P*	Crude HR (95% CI)	*P*	Adjusted HR (95% CI)	*P*
Dialysate Calcium Concentrations (vs.1.5 mmol/L)	0.74 (0.36,1.52)	0.41	1.01 (0.45,2.25)	0.98	0.43 (0.23,0.80)	**0.01**	0.47 (0.24,0.91)	**0.03**
Sex (vs. male)	1.16 (0.57,2.38)	0.68			1.12 (0.62,2.01)	0.71		
Age	1.04 (1.02,1.07)	**0.002**	1.01 (0.98,1.05)	0.49	1.05 (1.03,1.07)	**<0.001**	1.01 (0.99,1.04)	0.32
**Cause of ESRD**								
Glomerulus nephritis	Ref.		Ref.		Ref.			
Diabetic nephropathy	3.48 (1.66,7.33)	**0.001**	1.88 (0.84,4.20)	0.13	1.76 (0.93,3.22)	0.09		
Other or unknown	1.68 (0.38,7.51)	0.48	1.68 (0.35,8.01)	0.52	1.44 (0.44,4.75)	0.55		
Charlson comorbidities index	1.63 (1.35,1.97)	**<0.001**	1.29 (0.88,1.90)	0.19	1.70 (1.45,2.00)	**<0.001**	1.12 (0.83,1.51)	0.47
Cerebrovascular disease (vs.none)	4.53 (2.20,9.36)	**<0.001**	1.60 (0.61,4.18)	0.34	4.79 (2.65,8.66)	**<0.001**	2.56 (1.19,5.52)	**0.02**
Peripheral vascular disease (vs.none)	2.46 (1.19,5.09)	**0.02**	1.01 (0.40,2.51)	0.99	4.64 (2.57,8.37)	**<0.001**	2.28 (1.04,5.02)	**0.04**
Congestive heart failure (vs.none)	3.36 (1.57,7.19)	**0.002**	2.17 (0.93,5.06)	0.08	4.69 (2.53,8.68)	**<0.001**	2.97 (1.47,5.99)	**0.002**
Serum creatinine	0.10 (0.10,1.00)	**0.01**	1.00 (0.10,1.00)	0.62	1.00 (0.10,1.00)	**0.001**	1.00 (1.00,1.00)	0.34
Hemoglobin	0.97 (0.95,0.99)	**0.01**	0.99 (0.96,1.01)	0.28	0.98 (0.96,0.99)	**0.01**	0.98 (0.97,1.01)	0.19
Plasma albumin	0.90 (0.82,1.00)	0.05			0.24 (0.10,0.54)	**0.001**	0.71 (0.24,2.11)	0.55
Serum intact-parathyroid hormone	1.00 (1.00,1.00)	**0.03**	1.00 (1.00,1.00)	**0.02**	1.00 (1.00,1.00)	0.56		
**Medication use**								
Noncalcium binder (vs. none)					0.36 (0.18,0.74)	**0.01**	0.55 (0.24,1.30)	0.17
Calcium binder (vs. none)					0.91 (0.47,1.77)	0.78		
Vitamin D (vs. none)					0.49 (0.26,0.91)	**0.03**	1.27 (0.57,2.81)	0.56
Cinacalcet (vs. none)					0.40 (0.19,0.86)	**0.02**	0.76 (0.29,2.02)	0.58
Dialysate Calcium Concentrations*Serum iPTH	1.00 (1.00,1.00)	0.41			1.00 (1.00,1.00)	0.10		

HR, Hazard ratio; 95% Cl, 95% confidence interval; MACCEs, main adverse cardiovascular and cerebrovascular events; DCa, dialysate calcium concentration; iPTH: intact-parathyroid hormone.

Note: *p* < 0.05 was considered as statistically significant. Bold was for *p* < 0.05 in multivariate Cox regression analysis.

#### 3.3.2. MACCEs

As shown in Table 3, the cumulative rate of MACCEs was significantly lower in the low DCa group compared with the matched mid DCa group, at 7.3 per 100 patient-years versus 20.7 per 100 patient-years (*p* < 0.001). In the low DCa group, MACCEs included 7 episodes of heart failure, 2 strokes, 1 case of intracerebral hemorrhage, 1 myocardial infarction, and 16 other events. By contrast, in the matched mid DCa group, the MACCEs comprised 17 episodes of heart failure, 6 strokes, 6 cases of intracerebral hemorrhage, 1 myocardial infarction, and 45 other events.

**Figure 3.**
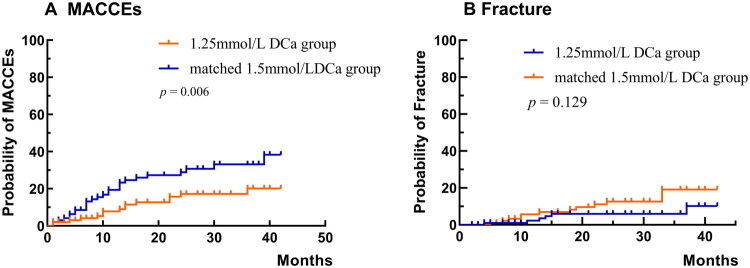
Kaplan-Meier MACCE estimates and fracture for the 1.25 mmol/L DCa group and matched 1.5 mmol/L DCa group patients. MACCEs, main adverse cardiovascular and cerebrovascular events; DCa: dialysate calcium.

The crude HR for the occurrence of the first MACCE in the low DCa group compared with the matched mid DCa group was 0.43 (95% CI: 0.23-0.80), and the adjusted HR was 0.47 (95% CI: 0.24-0.91) (Table 4). The Kaplan-Meier curve further demonstrated that the low DCa group was associated with a significantly lower risk of experiencing the first MACCE (log-rank *p* = 0.006, Figure 3A). The interaction between dialysis calcium concentration and iPTH was non-significant (HR1.00, 95%CI:1.00-1.00), suggesting no multiplicative effect of dialysis calcium concentration and iPTH on the first MACCE outcome (Table 4).

#### 3.3.3. Fracture

In the low DCa group, 6 patients (5.9%) experienced a cumulative fracture event compared with 14 patients (13.9%) in the matched mid DCa group. The cumulative fracture rates were 1.6 per 100 patient-years in the low DCa group and 3.9 per 100 patient-years in the matched mid DCa group (*p* = 0.003, Table 2).

The crude HR of the first fracture event in the low DCa group and the matched mid DCa group was 0.48 (95% CI: 0.18-1.27), and the adjusted HR was 0.66 (95% CI: 0.23-1.90) (Table 5). The Kaplan–Meier survival curve showed no significant difference in the incidence of the first fracture event between the two groups (log-rank *p* = 0.129, Figure 3B).

**Table 5.** Risk factors for the first hospitalization event and the occurrence of first fracture assessed by Cox regression model after matching.

	Fracture				Hospitalization			
	Univariable		Multivariable		Univariable		Multivariable	
Variables	Crude HR (95% CI)	*P*	Adjusted HR (95% CI)	*P*	Crude HR (95% CI)	*P*	Adjusted HR (95% CI)	*P*
Dialysate Calcium Concentrations (vs. 1.5 mmol/L)	0.48 (0.18,1.27)	0.14	0.66 (0.23,1.90)	0.44	0.66 (0.37,1.18)	0.16	0.70 (0.46,1.04)	0.08
Sex (vs. male)	1.46 (0.58,3.72)	0.42			1.07 (0.60,1.88)	0.83		
Age	1.02 (0.99,1.05)	0.22			1.02 (1.00,1.04)	**0.04**	1.01 (1.00,1.03)	0.20
**Cause of ESRD**								
Glomerulonephritis	Ref.		Ref.		Ref.			
Diabetic nephropathy	3.50 (1.30,9.40)	**0.01**	1.68 (0.33,8.65)	0.53	0.83 (0.41,1.67)	0.60		
Other or unknown	3.15 (0.65,15.18)	0.15	3.39 (0.60,19.12)	0.17	1.37 (0.42,4.47)	0.60		
Charlson comorbidities index	1.37 (1.04,1.80)	**0.02**	0.86 (0.56,1.31)	0.48	1.01 (0.82,1.25)	0.91		
**Comorbidities**								
Diabetes (vs.none)	3.44 (1.33,8.88)	**0.01**	2.91 (0.43,19.68)	0.27	1.13 (0.62,2.06)	0.70		
Coronary artery disease (vs.none)	3.27 (1.23,8.73)	**0.02**	3.03 (0.99,9.32)	0.05	1.66 (0.80,3.43)	0.17		
Cerebrovascular disease (vs.none)	2.13 (0.82,5.50)	0.12			1.68 (0.88,3.19)	0.11		
Peripheral vascular disease (vs.none)	1.20 (0.43,3.37)	0.73			0.87 (0.42,1.79)	0.70		
Congestive heart failure (vs.none)	3.42 (1.28,9.12)	**0.01**	3.16 (0.98,10.25)	0.06	1.00 (0.42,2.35)	0.10		
Serum creatinine	1.00 (1.00,1.00)	**0.02**	1.00 (1.00,1.00)	0.56	1.00 (1.00,1.00)	**0.03**	1.00 (1.00,1.00)	0.14
Hemoglobin	0.97 (0.94,0.99)	**0.02**	0.97 (0.94,1.00)	0.06	1.00 (0.98,1.02)	0.86		
Plasma albumin	0.25 (0.07,0.96)	**0.04**	0.62 (0.11,3.58)	0.60	0.50 (0.21,1.20)	0.12		
Serum intact-parathyroid hormone	1.00 (1.00,1.00)	0.06			1.00 (1.00,1.00)	**0.001**	1.00 (1.00,1.00)	**0.03**
**Medication use**								
Noncalcium binder (vs. none)	0.39 (0.13,1.19)	0.10			0.52 (0.34,0.80)	**0.003**	0.64 (0.41,1.01)	0.05
Calcium binder (vs. none)	0.96 (0.34,2.70)	0.94			0.87 (0.56,1.36)	0.55		
Vitamin D (vs. none)	0.46 (0.17,1.30)	0.15			0.93 (0.63,1.37)	0.71		
Cinacalcet (vs. none)	0.25 (0.06,1.09)	0.07			0.64 (0.41,0.99)	**0.04**	0.87 (0.54,1.41)	0.58

HR, Hazard ratio; 95% Cl, 95% confidence interval; DCa, dialysate calcium concentration.

Note: *p* < 0.05 was considered as statistically significant. Bold was for *p* < 0.05 in multivariate Cox regression analysis.

#### 3.3.4. Hospitalization

The cumulative hospitalization rates were 21.5 per 100 patient-years in the low DCa group compared with 34 per 100 patient-years in the matched mid DCa group, representing a statistically significant difference (*p* < 0.001, Table 2).

The crude HR of the first hospitalization event in the low DCa group and the matched medium DCa group was 0.66 (95% CI: 0.37-1.18), and the adjusted HR was 0.70 (95% CI: 0.46-1.04) (Table 5).

#### 3.3.5. Subgroup analyses by iPTH levels

**Figure 4.**
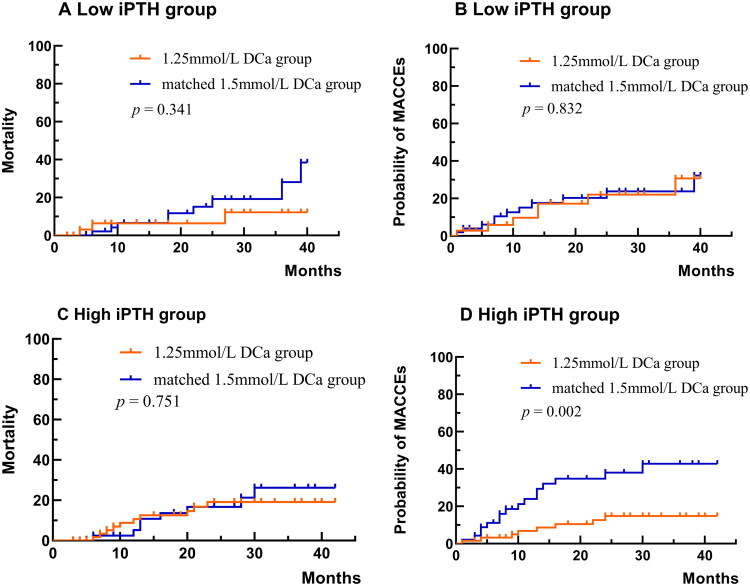
Kaplan-Meier Curves for mortality and MACCE estimates stratified by iPTH levels. A: Mortality in the low iPTH group; B: MACCEs probability in the low iPTH group; C: Mortality in the high iPTH group; D: MACCEs probability in the high iPTH group. MACCEs, main adverse cardiovascular and cerebrovascular events; DCa: dialysate calcium; iPTH: intact-parathyroid hormone.

Based on the interaction effect analysis, all matched-pair patients were stratified into two groups according to iPTH levels, using a cutoff value of 300 pg/mL. In the low iPTH group, neither univariable nor multivariable Cox proportional hazards analysis revealed a significant association between dialysis calcium concentration and all-cause mortality rate. In addition, neither univariable nor multivariable Cox proportional hazards analysis revealed a significant association between dialysis calcium concentration and the first MACCE. The interaction between dialysis calcium concentration and iPTH was non-significant, suggesting no multiplicative effect of dialysis calcium concentration and iPTH on mortality and the first MACCE outcome (Table 6). The Kaplan–Meier curve showed that in the low iPTH group, there was no significant difference in the all-cause mortality rate (log-rank test *p* = 0.341, Figure 4A) and the first MACCE event (log-rank test *p* = 0.832, Figure 4B) between the two groups.

**Table 6.** Risk factors for mortality and the occurrence of first MACCE assessed by Cox regression model in low iPTH group.

	Death				MACCEs			
	Univariable		Multivariable		Univariable		Multivariable	
Variables	Crude HR (95% CI)	*P*	Adjusted HR (95% CI)	*P*	Crude HR (95% CI)	*P*	Adjusted HR (95% CI)	*P*
Dialysate Calcium Concentrations (vs. 1.5 mmol/L)	0.54 (0.14,1.98)	0.35	2.20 (0.40,12.01)	0.36	0.72 (0.29,1.77)	0.50	0.73 (0.26,2.06)	0.55
Sex (vs. male)	1.75 (0.56,5.43)	0.34			0.62 (0.24,1.60)	0.32		
Age	1.03 (0.99,1.08)	0.13			1.03 (1.00,1.07)	**0.04**	1.00 (0.96,1.04)	0.90
**Cause of ESRD**								
Glomerulonephritis	Ref.				Ref.			
Diabetic nephropathy	3.41 (1.04,11.21)	**0.04**			1.42 (0.51,3.94)	0.50		
Other or unknown	2.22 (0.26,18.57)	0.46			2.61 (0.47,9.16)	0.34		
Charlson comorbidities index	1.60 (1.21,2.11)	**0.001**	1.67 (0.99,2.82)	0.05	1.49 (1.20,1.85)	**<0.001**	0.98 (0.67,1.45)	0.99
Cerebrovascular disease (vs.none)	3.92 (1.26,12.17)	**0.02**	0.66 (0.14,3.15)	0.60	5.17 (2.18,12.27)	**<0.001**	2.67 (0.80,8.97)	0.11
Peripheral vascular disease (vs.none)	4.42 (1.40,13.97)	**0.01**	3.60 (0.79,16.52)	0.10	4.70 (1.98,11.16)	**<0.001**	3.92 (1.22,12.58)	**0.02**
Congestive heart failure (vs.none)	3.50 (1.11,11.07)	**0.03**	0.93 (0.22,3.89)	0.92	3.96 (1.63,9.62)	**0.002**	1.99 (0.71,5.59)	0.19
Serum creatinine	1.00 (1.00,1.00)	**0.02**	1.00 (1.00,1.00)	0.22	1.00 (1.00,1.00)	**0.03**	1.00 (1.00,1.00)	0.01
Hemoglobin	0.96 (0.93,0.99)	**0.002**	0.97 (0.94,1.01)	0.12	0.98 (0.95,1.00)	**0.03**	0.98 (0.96,1.01)	0.21
Plasma albumin	0.05 (0.01,0.38)	**0.003**	0.18 (0.01,2.48)	0.20	0.29 (0.07,1.22)	0.09		
Serum intact PTH	1.00 (1.00,1.01)	0.66			1.00 (1.00,1.01)	0.24		
Dialysate Calcium Concentrations*Serum iPTH	1.00 (0.99,1.00)	0.34			1.00 (0.99,1.00)	0.47		

HR, Hazard ratio; 95% Cl, 95% confidence interval; MACCEs, main adverse cardiovascular and cerebrovascular events; DCa, dialysate calcium concentration. iPTH: intact-parathyroid hormone.

Note: *p* < 0.05 was considered as statistically significant. Bold was for *p* < 0.05 in multivariate Cox regression analysis.

In the high iPTH group, neither univariable nor multivariable Cox proportional hazards analysis revealed a significant association between dialysis calcium concentration and all-cause mortality rate. However, the crude HR for the occurrence of the first MACCE in the low DCa group compared with the matched mid DCa group was 0.29 (95% CI: 0.13-0.68), and the adjusted HR was 0.27 (95% CI: 0.10-0.68). The interaction between dialysis calcium concentration and iPTH was non-significant, suggesting no multiplicative effect of dialysis calcium concentration and iPTH on mortality and the first MACCE outcome (Table 7). The Kaplan-Meier curve further demonstrated that in the high iPTH group, the risk of the first MACCE was significantly lower in the low DCa group (log-rank test *p* = 0.002, Figure 4D), which was consistent with the results before the subgroup analysis.

**Table 7.** Risk factors for mortality and the occurrence of first MACCE assessed by Cox regression model in high iPTH group.

	Death				MACCEs			
	Univariable		Multivariable		Univariable		Multivariable	
Variables	Crude HR (95% CI)	*P*	Adjusted HR (95% CI)	*P*	Crude HR (95% CI)	*P*	Adjusted HR (95% CI)	*P*
Dialysate Calcium Concentrations (vs. 1.5 mmol/L)	0.86 (0.34,2.18)	0.75	1.47 (0.51,4.26)	0.48	0.29 (0.13,0.68)	**0.01**	0.27 (0.10,0.68)	**0.01**
Age	1.05 (1.01,1.08)	**0.01**	1.01 (0.97,1.01)	0.51	1.51 (1.03,1.09)	**<0.001**	1.03 (0.99,1.07)	0.17
**Cause of ESRD**								
Glomerulonephritis	Ref.				Ref.			
Diabetic nephropathy	3.45 (1.31,9.07)	**0.01**	1.97 (0.67,5.78)	0.22	1.98 (0.87,4.49)	0.10		
Other or unknown	1.48 (0.18,12.04)	0.71	2.23 (0.24,20.46)	0.48	0.93 (0.12,7.13)	0.94		
Charlson comorbidities index	1.80 (1.29,2.51)	**0.001**	1.33 (0.77,2.30)	0.31	2.50 (1.76,3.54)	**<0.001**	1.38 (0.81,2.34)	0.24
Cerebrovascular disease (vs.none)	4.75 (1.84,12.25)	**0.001**	2.03 (0.51,8.00)	0.31	4.76 (2.09,10.85)	**<0.001**	2.82 (0.97,8.21)	0.06
Peripheral vascular disease (vs.none)	1.49 (0.60,3.98)	0.42			4.54 (2.03,10.19)	**<0.001**	2.32 (0.79,6.81)	0.13
Congestive heart failure (vs.none)	3.40 (1.21,9.59)	**0.02**	2.31 (0.72,7.37)	0.16	5.60 (2.38,13.20)	**<0.001**	5.44 (1.80,16.47)	**0.003**
Serum creatinine	1.00 (1.00,1.00)	0.15			1.00 (1.00,1.00)	**0.01**	1.00 (1.00,1.00)	0.98
Plasma albumin	0.84 (0.23,3.11)	0.80			0.18 (0.06,0.52)	**0.001**	0.37 (0.09,1.47)	0.16
Serum intact-parathyroid hormone	1.00 (1.00,1.00)	**0.001**	1.00 (1.00,1.00)	**0.003**	1.00 (1.00,1.00)	0.33		
Dialysate Calcium Concentrations*Serum iPTH	1.00 (1.00,1.00)	0.34			1.00 (1.00,1.00)	0.12		

HR, Hazard ratio; 95% Cl, 95% confidence interval; MACCEs, main adverse cardiovascular and cerebrovascular events; DCa, dialysate calcium concentration. iPTH: intact-parathyroid hormone.

Note: *p* < 0.05 was considered as statistically significant. Bold was for *p* < 0.05 in multivariate Cox regression analysis.

## 4. Discussion

In this retrospective observational cohort study, we compared the impact of DCa concentration on clinical outcomes in HD patients treated between January 1, 2019, and December 31, 2023. Our findings showed no significant difference in mortality between low DCa and matched mid DCa patients following matched-pair analysis. However, within the matched-pair cohort, patients receiving low DCa had a lower risk of developing MACCEs, a reduced cumulative incidence of fractures, and lower hospitalization rates compared with those receiving mid DCa. These results provide new evidence supporting the potential benefits of low DCa in clinical practice.

The regulation of calcium balance in HD patients is highly complex, and the optimal dialysis calcium concentration has yet to be clearly defined. CKD-MBD is a significant complication of ESRD, closely linked to both all-cause mortality and cardiovascular mortality. Treatment for CKD-MBD typically involves lowering elevated phosphorus levels while maintaining calcium within normal ranges. DCa concentration plays a crucial role in helping to maintain calcium balance in MHD patients. However, achieving and maintaining this balance remains a significant challenge. Previous studies have shown that low DCa (1.25 mmol/L) is associated with an increased risk of arrhythmia and sudden death in HD patients [16]. By contrast, higher DCa levels can lead to more stable hemodynamics and significant reductions in serum phosphorus and iPTH concentrations. However, over the long term, higher DCa levels may contribute to the development of cardiovascular calcification and nephrogenic osteodystrophy [17].

Our study found no statistically significant difference in survival rates between the low DCa group and the matched mid DCa group after matching, which aligns with the findings of a previous study [18]. In that cohort study conducted in France, variations in dialysis calcium concentration (1.25, 1.5, or 1.75 mmol/L) did not significantly impact the 42-month survival rate. This lack of significant findings in our study may be attributed to the relatively short observation period of our cohort. Our center has only collected data over a 48-month period, and the limited duration of follow-up may explain the absence of notable differences in mortality rates.

This study examined the relationship between dialysis calcium concentration and hospitalization rates. However, due to challenges in data collection, we were unable to determine the specific causes of readmission. Despite this limitation, our findings indicate that the cumulative hospitalization rate was significantly higher in the matched mid DCa group compared with the low DCa group [7]. Although we did not report the specific reasons for hospitalization, previous studies have shown that hospitalization rates for cardiovascular and infectious disease-related conditions are higher in the high DCa group than in the low DCa group. Studies have linked elevated calcium levels to accelerated cell apoptosis, reduced phagocytic activity, and weakened oxidative responses in neutrophils, which may increase infection-related complications and mortality in dialysis patients [19,20]. However, our study did not find a higher incidence of infectious diseases in the low DCa group compared with the matched mid DCa group. This could be due to the smaller number of patients in the matched cohort. In addition, another study found that reducing the calcium concentration in dialysis fluid from 2.5 mEq/L (1.5 mmol/L) to a lower level worsened the relationship between heart failure and increased hospitalization [9]. Our data showed a higher incidence of MACCEs in the matched mid DCa group, suggesting that variations in calcium concentration during dialysis may influence the incidence of cardiovascular events. Both high and low calcium levels may increase MACCE rates, a key factor contributing to mortality and hospitalization.

Our data show that in the matched cohort, the cumulative incidence of MACCEs was significantly lower in the low DCa group compared with the matched mid DCa group. However, previous studies have yielded inconsistent conclusions regarding the impact of dialysis calcium concentration on cardiovascular events. Some studies suggest that low DCa may be beneficial in preventing arterial calcification or atherosclerosis in various vascular territories, potentially reducing the incidence of MACCEs [21–23]. By contrast, other research has shown that low DCa could increase coronary calcification [24]. We propose that low-calcium dialysate helps lower blood calcium levels in dialysis patients, while simultaneously increasing iPTH levels. This facilitates calcium deposition in bones, reducing the risk of calcium accumulation in soft tissues (such as blood vessels) and thus preventing cardiovascular events. In addition, the low-calcium environment may inhibit the phenotypic transformation and calcification of vascular smooth muscle cells, while enhancing endothelial function.

Our baseline data indicated that the median iPTH and serum phosphorus levels were higher in the low DCa group compared with the matched mid DCa group. Although these data were obtained from patients’ pre-dialysis information, they reflect the long-term effects of stable treatment. Previous studies have shown a significant association between elevated phosphorus levels and secondary hyperparathyroidism with the incidence of cardiovascular events and fractures. In addition, increasing DCa has been shown to effectively regulate serum iPTH and phosphorus concentrations [1,25]. However, our findings differ from these conclusions. Our data suggest that the cumulative incidence of MACCEs, along with the results from Cox regression analysis and the Kaplan–Meier curve for the first MACCE occurrence, indicates that the risk of MACCEs in the low DCa group is lower than that in the matched mid DCa group. We hypothesize that this may be due to increasing the calcium concentration in the dialysate, which raises the body’s calcium load. In Asia, HD patients often use calcium salts (such as calcium carbonate and calcium acetate) and vitamin D3 agents (including calcitriol and alpha-hydroxyvitamin D3), potentially leading to a positive calcium balance. This condition may exacerbate hypercalcemia, cause low bone turnover rates, and contribute to coronary artery calcification, ultimately increasing the fracture risk over time. A prospective clinical study demonstrated that in HD patients with baseline parathyroid hormone levels up to 300 pg/mL, reducing the calcium concentration in the dialysate could slow coronary calcification progression and improve bone metabolism. This finding further supports our hypothesis [22].

This study revealed an unexpected finding: although the Kaplan–Meier survival curve showed no significant difference between the low DCa and matched mid DCa groups for the first fracture incidence, the cumulative incidence of fractures was lower in the low DCa group. It is commonly believed that low DCa may induce hypocalcemia, which could increase the risk of fracture events. However, numerous studies suggest that low DCa can elevate parathyroid hormone and bone formation marker levels (such as alkaline phosphatase), enhance bone turnover, and thereby reduce fracture risk. Based on these findings, we propose that using low DCa may not increase the fracture risk in HD patients and could even improve nephrogenic osteodystrophy to some extent [26–28]. Nevertheless, since the first and cumulative fracture event rates were not significantly different in our study, we did not perform further multivariable analysis. We hope that future research will more thoroughly investigate the relationship between dialysis calcium concentration and fracture risk.

This study has several limitations. The primary limitation is its retrospective cohort design, rather than a randomized controlled trial. Although we matched and adjusted for various confounding factors, residual confounding may have still existed, potentially introducing bias. Additionally, this study was conducted at a single center with a relatively small sample size, and we were unable to implement a 1:2 matched-pair analysis, which could have better represented the data. It should be noted that the number of outcome events in this study was indeed relatively small in both groups after matching, which may compromise the stability of the multivariate model. Nevertheless, our findings still indicate that patients in the low Dca group have a lower risk of developing MACCEs. In future studies, extending the follow-up period and validating the results through larger-sample investigations could enhance the robustness of the conclusions. Full baseline comparability was also not achieved, which could affect the results, though we attempted to minimize this bias through stratified analysis. Furthermore, only baseline laboratory parameter results were included in this study, and these values may have fluctuated during the course of dialysis treatment. As such, the laboratory data presented in this study may not fully reflect the patients’ conditions throughout the treatment period. Additionally, critical data on vascular calcification, bone turnover markers, ionized calcium levels, overall calcium balance, and vitamin D levels—factors that could provide insight into the mechanisms underlying the relationship between DCa and mortality-were not available. Finally, we could not investigate the impact of calcium dialysis duration on the outcome further. Although this is a notable limitation, it should be emphasized that most dialysis centers and patients generally maintain a consistent DCa concentration over extended periods.

## 5. Conclusion

In conclusion, our study revealed no significant difference in all-cause mortality between MHD patients treated with low DCa and those using matched mid DCa. However, significant differences were observed in the incidence of MACCEs, cumulative fracture incidence, and hospitalization rates between the two groups. While DCa prescriptions should be tailored to the individualized needs of each MHD patient, our findings cautiously suggest that the use of low DCa may contribute to reducing the incidence of MACCEs, hospitalizations, and fractures.

## Data Availability

The datasets used and/or analyzed in this study are available from the corresponding author upon reasonable request and with approval from the Zhongshan Hospital of Traditional Chinese Medicine Affiliated to Guangzhou University of Traditional Chinese Medicine.
